# Characterization and evaluation of a femtosecond laser-induced osseointegration and an anti-inflammatory structure generated on a titanium alloy

**DOI:** 10.1093/rb/rbab006

**Published:** 2021-03-13

**Authors:** Yang Liu, Zhongying Rui, Wei Cheng, Licheng Song, Yunqiang Xu, Ruixin Li, Xizheng Zhang

**Affiliations:** 1 Department of Orthopedics, Tianjin Medical University General Hospital, Tianjin 300052, China; 2 Institute of Medical Service Support, Institute of Systems Engineering, Academy of Military Sciences, Tianjin, China; 3 Department of Nuclear Medicine, Tianjin Medical University General Hospital, Tianjin 300052, China; 4 Tianjin Key Laboratory of Oral and Maxillofacial Function Reconstruction, Tianjin Stomatological Hospital, The Affiliated Stomatological Hospital of Nankai University, Tianjin 300041, China

**Keywords:** femtosecond laser, hybrid micro-/nanostructure, cell–material interactions, osseointegration, macrophage polarization

## Abstract

Cell–material interactions during early osseointegration of the bone–implant interface are critical and involve crosstalk between osteoblasts and osteoclasts. The surface properties of titanium implants also play a critical role in cell–material interactions. In this study, femtosecond laser treatment and sandblasting were used to alter the surface morphology, roughness and wettability of a titanium alloy. Osteoblasts and osteoclasts were then cultured on the resulting titanium alloy disks. Four disk groups were tested: a polished titanium alloy (pTi) control; a hydrophilic micro-dislocation titanium alloy (sandblasted Ti (STi)); a hydrophobic nano-mastoid Ti alloy (femtosecond laser-treated Ti (FTi)); and a hydrophilic hierarchical hybrid micro-/nanostructured Ti alloy [femtosecond laser-treated and sandblasted Ti (FSTi)]. The titanium surface treated by the femtosecond laser and sandblasting showed higher biomineralization activity and lower cytotoxicity in simulated body fluid and lactate dehydrogenase assays. Compared to the control surface, the multifunctional titanium surface induced a better cellular response in terms of proliferation, differentiation, mineralization and collagen secretion. Further investigation of macrophage polarization revealed that increased anti-inflammatory factor secretion and decreased proinflammatory factor secretion occurred in the early response of macrophages. Based on the above results, the synergistic effect of the surface properties produced an excellent cellular response at the bone–implant interface, which was mainly reflected by the promotion of early ossteointegration and macrophage polarization.

## Introduction

Titanium alloy (Ti6Al4V) implants are widely used in the clinic because of their high-specific strength, low modulus of elasticity, strong corrosion resistance and good biocompatibility. However, the biological binding stability of titanium implants has not yet reached a satisfactory level. The contact surface instability at the implant interface is affected by many factors that can lead to mechanical damage, aseptic loosening, instability and fracture. Eventually, the prosthetic implant may detach and become ectopic, which usually leads to revision surgery [1, 2]. Slow early osseointegration and local inflammatory responses have a significant effect on the failure of titanium alloy implants. Thus, for the long-term safety of patients, the osteogenic stability and anti-inflammatory properties of titanium implants are crucial.

The cell–material interactions of osteoblasts and osteoclasts at the interface of the implant depend on the physicochemical properties of the implant surface, including the surface topography, roughness, wettability and chemical composition. For osteoblasts, studies have shown that a micro-/nanostructured with a certain roughness can retain the beneficial properties of the substrate and improve implant osseointegration, resulting in a stronger bond between the implant and tissue [3–7]. Pores in the surface and increased hydrophilicity have been proven to have a positive effect on the early ossteointegration of osteoblasts [8–10]. Meanwhile, the unique surface structure and ligand landscape have different effects on the apparent activation of macrophages [11–13]. M1/M2 macrophage activation and the corresponding released factors regulate the microenvironment around implants in response to their interaction with the surface of biomaterials, thereby controlling the immune response [14]. Various studies have focused on enhancing osteoblast compatibility or macrophage activation rather than their synergistic properties.

Several methods have been effectively used to change the surface performance of materials, such as anodic oxidization [15], plasma spraying [16, 17], magnetron sputtering [18] and mechanical grinding [19]. These randomized processes create a coarse roughness, which is unstructured and uncontrolled, at the implant surface [20]. Femtosecond laser texturing is a promising biomaterial processing technology. Its advantages include wide applicability to most types of materials [21]. the possibility of obtaining a wide variety of structures at the nano- and microscale, high resolution and fast, repeatable and contactless processing [22]. In addition, the sandblasting process can quickly create a rough surface and improve the interfacial bonding force without adding other elemental impurities [23].

Based on the functional plasticity of osteoblasts and osteoclasts, this study had two aims. First, we aimed to obtain a suitable roughness and primary structure by sandblasting. The secondary surface morphology and beneficial wettability were achieved through femtosecond laser processing. Next, by adjusting the surface properties of the titanium alloy substrate, we developed a clinically relevant dual-function surface modification process that was able to modify the early surface osseointegration and anti-inflammatory properties. Second, we aimed to evaluate the effect of this modified micro-/nanocomposite surface morphology and investigate the surface activity of the materials *in vitro* regarding bone growth, osseointegration and macrophage polarization.

## Materials and methods

### Specimen preparation

Commercial titanium alloy disks (10 mm in diameter, 0.8 mm in thickness) were polished with SiC paper of varying grit (#360, #600, #1000, #2000, Matador, Germany). The polished titanium alloy (pTi) substrate was thoroughly cleaned by continuous ultrasonic treatment in acetone and isopropanol and then rinsed in deionized water. Al_2_O_3_ with a 90 μm particle size was sprayed 100 mm away from the surface of the specimen at a spray pressure of 0.5 MPa and an angle of 45°. The sandblasted titanium alloy (STi) disks were then ultrasonically washed in 60°C deionized water for one hour. Residual impurities on the surface were removed. A high-energy, industrial, integrated, ultrafast amplification laser system (Solstice Ace, Spectra-Physics) was used for surface microfabrication. The laser provides pulses with a center wavelength of 800 nm, a pulse width of 100 fs, a repetition rate of 1 kHz, a beam diameter (1/e^2^) of 30 mm, a high pulse energy of 6 mJ and a laser energy fluence of 6.7 J/cm^2^. A combination of a 1/2 wave plate and a polarizer was employed to continuously vary the laser energy. The average laser power was measured by a power meter (843-R, Newport). The diameter of the laser beam on the sample was estimated as approximately 25 μm after focusing with a scanning galvanometer (IntelliSCAN III 14, SCANLAB) [24]. After femtosecond laser treatment, the polished titanium alloy and sandblasted titanium alloy formed the femtosecond laser-treated titanium alloy (FTi) group and the micro-/nanostructured surface (FSTi) group, respectively. The ultrasonic washing process was then repeated. The samples were stored in vacuum packages after treatment. We obtained specimens with a specific roughness and surface morphology, which we characterized by a series of methods to explain the differences in surface properties. The precise mechanisms by which the laser treatment and sandblasting affected the surface properties are beyond the scope of this study and will not be discussed in detail here. However, we investigated these key parameters in detail and established experimental repeatability. Samples were stored in vacuum packages after treatment. We obtained the specimens with specific roughness and surface morphology, and took a series of characterization to explain the difference of surface properties.

### Surface characterization

The surface morphology of the samples was examined using a field-emission scanning electron microscope (Hitachi SU8100, Japan). The surface roughness was measured by a roughness meter (Marsurf PS10, Germany); the average surface roughness (Ra) was selected as the depth parameter. For phase identification, the samples were first examined by X-ray diffractometry (XRD, PANalytical XPert PRO, Holland) at 40 kV and 100 mA. Phase identification was performed using the standard International Centre for Diffraction Data (ICDD) database. Three-dimensional imaging and the quantification of surface topographic features were performed using an atomic force microscope (NanoWizard, JPK Instruments). Topographical images of the surface of substrate samples 5 × 5 mm^2^ in size were obtained in tapping mode. The surface wettability was determined by the drop contact angle method (DSA100, DataPhysics Instruments GmbH, Flindern, Germany). A 2 µL water droplet was placed on the surface of each sample, and the contact angle was measured for 3 s. The operation was repeated three times.

### Bioactivity *in vitro*

All of the titanium alloy samples were incubated in simulated body fluid (SBF, Solarbio, China) at 37°C, and apatite deposition was evaluated *in vitro*. SBF was prepared based on the method reported by Kokubo [25]. After incubation in SBF for 14 days, the samples were removed, rinsed with distilled water and air-dried. The amount of apatite deposited on the titanium alloy disk surfaces was determined by SEM (Hitachi SU8100, Japan) and XRD (PANalytical XPert PRO, Holland).

### Protein adsorption assay

Bovine serum albumin (BSA, Solarbio, China) was used as a model protein. One milliliter of protein solution (1 mg/mL BSA/phosphate-buffered saline (PBS)) was pipetted onto the titanium surfaces. After 3 h of incubation at 37°C, the samples were moved into a 24-well plate and washed with PBS （Solarbio, China). Nonadherent proteins were removed, and the rest of the proteins were dissolved in 600 μL of 1% sodium dodecyl sulfate (SDS, Solarbio, China). The protein content in the SDS solution was determined using a bicinchoninic acid (BCA) protein assay kit (Applygen Technologies, Inc., China). The test results were normalized by a BCA standard protein curve.

### *In vitro* cell culture

The mouse MC3T3-E1 preosteoblastic cell line (National Infrastructure of Cell Line Resources, China) was used in the biological assays. Briefly, osteoblasts were seeded in alpha-modified Gibco minimum essential medium (Gibco, South America) supplemented with 10% fetal bovine serum (FBS), 50 μg/mL ascorbic acid, 10 mM Na-β-glycerophosphate, 10^−8^ M dexamethasone and antibiotic-antimycotic solution. Osteoblasts were incubated at 37°C in a humidified environment of 95% air and 5% CO_2_. When the cells reached 80% confluence, they were separated by 0.25% trypsin-ethylene diamine tetraacetic acid (EDTA, Solarbio, China). The culture medium was renewed every 3 days.

RAW 264.7 cells (National Infrastructure of Cell Line Resources, China) were cultured in Dulbecco’s modified Eagle’s medium (DMEM, Gibco, South America) supplemented with 10% FBS. Cells were seeded at a density of 1  ×  10^5^ cells/cm^2^ and cultured until reaching 80% confluency before being passaged with a plastic scraper (Greiner BioOne, Netherlands). The RAW 264.7 cells were incubated in a humidified atmosphere of 95% air and 5% CO_2_ at 37°C. The culture medium was renewed every 3 days.

### Cytotoxicity testing

Cells were seeded on four sets of titanium alloy samples at a density of 2  ×  10^3^/cm^2^. MC3T3-E1 preosteoblastic cells were exposed to four types of titanium alloy disks for 24 and 72 h to evaluate their cytotoxicity. After each incubation period, samples of the media were transferred into a fresh 96-well plate and analyzed for lactate dehydrogenase (LDH). The cytotoxicity assay was performed using an LDH Cytotoxicity Kit II (Jiancheng Bioengineering Institute, China), strictly following the manufacturer’s instructions.

### Cell attachment and proliferation assays

The initial attachment of cells to and proliferation of cells on the titanium disks was monitored by Cell Counting Kit-8 (CCK-8, Dojindo Laboratories, Kumamoto, Japan) assay. Typically, 2 × 10^3^ cells were seeded into 24-well plates before conducting the experiment. The cells were cultured for 4 h, 1, 3, 5 and 7 days. The culture medium was renewed every 3 days. After the specified incubation periods, 50 µL of 2-(2-methoxy-4-nitrophenyl)-3-(4-nitrophenyl)-5-(2,4-disulfophenyl)-2H-tetrazoliumsodiumsalt solution was added to each well, followed by incubation at 37°C for 3 h. The background absorbance was subtracted from the formazan absorbance at each time point. The absorbance at 450 nm was determined using a microplate reader (Tecan Infinite^®^ F500, Switzerland).

### Extracellular alkaline phosphatase (ALP) activity

The level of extracellular ALP produced by the MC3T3-E1 cells was measured to assess the functional activity of cells cultured on each sample in normal media and under standard conditions. Cells were cultured for 7 and 14 days at a density of 1 × 10^4^ cells per sample. After culturing, the cells were evaluated using an ALP activity kit (Jiancheng Bioengineering Institute, China). The cells incubated on the titanium alloy disks were washed three times with PBS and then 500 μL of 0.1% Triton X-100 was applied to each well. The absorbance at 450 nm was measured using a microplate reader. The test results were normalized by a BCA standard protein curve. ALP activity is expressed as the micromolar concentration of p-nitrophenol produced per microgram protein per minute (μmol min^−1^/μg protein).

### Cell morphology

Aliquots of 2 × 10^3^ MC3T3-E1 preosteoblasts were seeded on four types of titanium alloy samples with different surface morphologies. After 9 h of culture, the osteoblasts were rinsed with PBS and fixed in 2.5% glutaraldehyde overnight. The cells were then dehydrated in 30%, 50%, 60%, 70%, 80% and 90% ethanol (Solarbio, China) for 10 min each. After dehydration, the samples were dried in a critical point carbon dioxide dryer and then sputter-coated with gold. The surface of the specimens was observed and imaged at various locations and magnifications by SEM (FESEM; Hitachi SU8100, Japan).

### Extracellular matrix (ECM) mineralization assay

The cell culture method was the same as described above. After 21 days of culture, the cells were washed with PBS and fixed for 4 h with 2.5% glutaraldehyde (Aladdin, China). After washing with distilled water, the osteoblasts were stained with 1 wt% alizarin red (Solarbio, China) for 5 min. The samples were rinsed three times with distilled water, and images were acquired by optical microscopy (Olympus U-P03, Japan) for qualitative analysis. For quantification, a 10% (v/v) acetic acid solution (Solarbio, China) was added to each well and then shaken, mixed and incubated for 30 min, followed by heating in a water bath at 85°C for 10 min. After the samples were centrifuged, 200 μL of the supernatant was neutralized with 200 μL of 10% tetramethylammonium hydroxide (Aladdin, China). The absorbance at 405 nm was measured using a microplate reader.

### Collagen secretion

The collagen secretion ability was qualitatively and quantitatively detected by sirius red (Solarbio, China). Cells were seeded in 24-well microplates at 2 × 10^3^ cells per well in normal media and cultured under standard conditions for 7 and 14 days. Next, the disks were washed with PBS, and 1 ml of 4% paraformaldehyde was added dropwise to the surface of the samples, followed by incubation for 4 h. Each well was stained with 0.1 wt% sirius red for 18 hours at room temperature; then 0.1 M acetic acid (Aladdin, China) was used to decolorize the titanium surface. Next, collagen secretion was imaged by an optical microscope (Olympus U-P03, Japan) for qualitative analysis. For quantification, the stain on the specimens was eluted in 500 μL of destaining solution (0.2 M NaOH/methanol 1:1, Aladdin, China). The optical density at 540 nm was then measured using a spectrophotometer [26].

### Macrophage polarization

Mouse RAW 264.7 macrophage-like cells were seeded at a density of 1 × 10^5^ cells per sample on four sets of titanium alloy samples with different surface topographies. RAW 264.7 cell cultures were established, as described previously. After 1 and 3 days of culture, the supernatant was collected and centrifuged for testing. The levels of IL-1β, IL-4, IL-6 and IL-10 in the conditioned media were measured by ELISA kits (Jiangsu Jingmei Biological Technology Co., Ltd, China) following the manufacturer’s protocol.

### 2.13. Statistical analysis

Each experiment was repeated three times. The data were obtained in at least triplicate and are presented as the mean ± SD of three independent experiments (*n* = 3) to ensure the accuracy of the experimental data. Analysis of variance (ANOVA) followed by the Bonferroni *post hoc* test was used to evaluate differences among groups. A *P* values of less than 0.05 was considered statistically significant. The statistical analyses were performed using IBM SPSS 22.0 (IBM Corp., version 22.0, Armonk, NY).

## Results

### Material characterization

**Figure 1.**
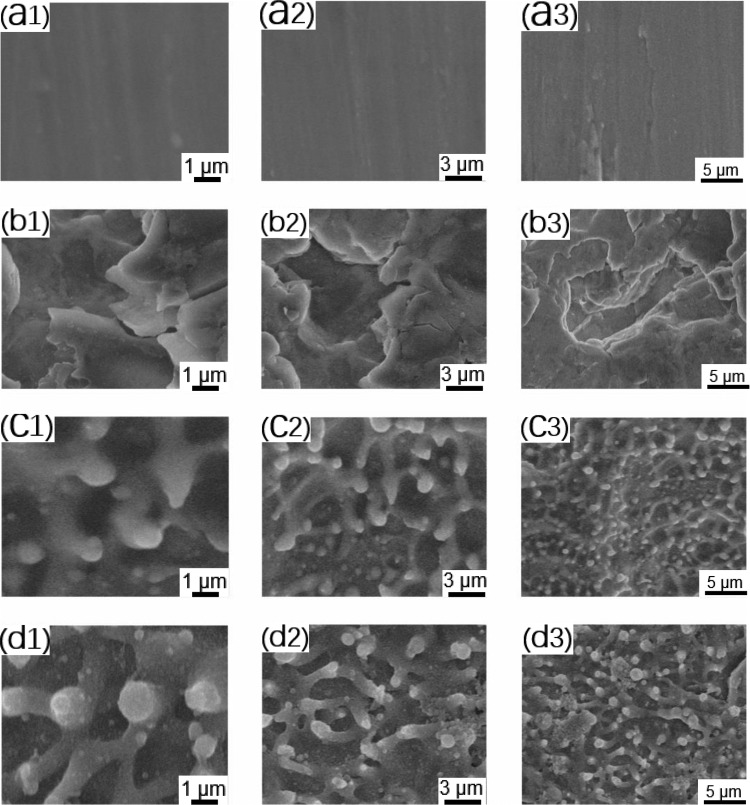
Representative SEM images of the different polished, sandblasted, femtosecond laser-treated and sandblasted + femtosecond laser-treated titanium disk surfaces used in the preliminary experiments: (**A**) polished titanium (pTi) at different magnifications; (**B**) sandblasted titanium (STi) at different magnifications; (**C**) femtosecond laser-treated titanium (FTi) at different magnifications; (**D**) femtosecond laser-treated and sandblasted titanium (FSTi) at different magnifications. The second and third columns in each group are low-magnification images of the samples.

SEM, atomic force microscopy and surface roughness analysis were used to characterize the structure of the titanium samples with different surface parameters after processing. The results were used to define the surface roughness of the titanium alloy samples. We followed the standards of Albrektsson [27], as follows: smooth surface (Ra: 0–0.4 μm), minimally rough surface (Ra: 0.5–1.0 μm), moderately rough surface (Ra: 1.0–2.0 μm) and rough surface (Ra: >2 μm). As shown in Fig. 1A (a1–a3), there were a few scratches on the surface of the polished titanium alloy (pTi). The Ra of the pTi was 0.07 ± 0.012 μm, which meets the standard of a smooth surface. The sandblasted surface (STi: Fig. 1B (b1–b3)) presented irregular, dislocated structures. The roughness of the sample was 3.77 ± 0.29 μm. The measured roughness value indicated that the sample had a rough surface. The Ra of the FTi was 0.56 ± 0.04 μm, which met the standard of a minimally rough surface. The surface of the femtosecond laser-treated titanium alloy (FTi: Fig. 1C (c1–c3)) showed a mastoid columnar structure and irregular holes distributed in the basal part of the titanium surface. SEM images of samples in the micro-/nanostructure group (FSTi: Fig. 1D (d1–d3)) revealed mastoid columns on the surface of the dislocated structures, along with a nonuniform distribution of interconnected pores. This biomimetic structure appeared to be similar to that of cancellous bone. The mean roughness was 3.83 ± 0.20 μm, indicating a rough surface. Notably, some particles melted on the surface treated by the femtosecond laser. Based on the properties of the femtosecond laser itself, rapid, uneven heating will cause ablation pits to form while vigorously spattering a large amount of burning debris and scattering it in the heat-affected area [28]. This is an inevitable by-product of the laser linear scanning mode.

### Wettability assay

**Figure 2.**
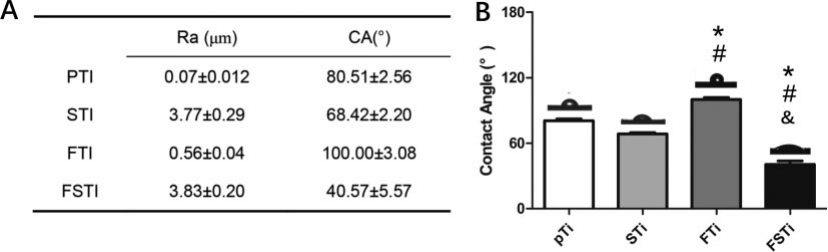
Characterization of the titanium surfaces. (**A**) Roughness and wettability of the titanium alloy substrates. (**B**) Contact angle of samples in the pTi, STi, FTi and FSTi groups. pTi: polished titanium; STi: sandblasted titanium; FTi: femtosecond laser-treated titanium; FSTi: femtosecond laser-treated and sandblasted titanium. Significance: *P <* *0.05 (STi, FTi, FSTi vs pTi.); ^#^*P *<* *0.05 (pTi, FTi, FSTi vs STi); ^&^*P *<* *0.05 (pTi, STi, FSTi vs FTi).

The static water contact angle is a common parameter used to understand the influence of sandblasting and femtosecond laser treatment on the wettability of titanium alloy surfaces. In general, if the static water contact angle is larger than 90°, the surface is regarded as hydrophobic. Otherwise, the surface is considered hydrophilic [29]. As shown in Fig. 2B, the average static contact angle of the polished titanium alloy (pTi) before sandblasting was 80.51 ± 2.56°, while the average contact angle of the sandblasted surface (STi) was 68.42 ± 2.20°. There were no significant differences between the pTi and STi groups (*P > *0.05) (supporting information). The contact angle of the surface treated by the femtosecond laser was approximately 100.00 ± 3.08°, suggesting that the disk was hydrophobic. There was no significant difference among the pTi, STi and FSTi groups (*P *<* *0.05). The micro-/nanostructured surface (FSTi) was the most hydrophilic material, with an average water contact angle of 40.57 ± 5.57°. This water contact angle was significantly different from that in the other three groups (*P *<* *0.05).

### Lattice structure assay

**Figure 3.**
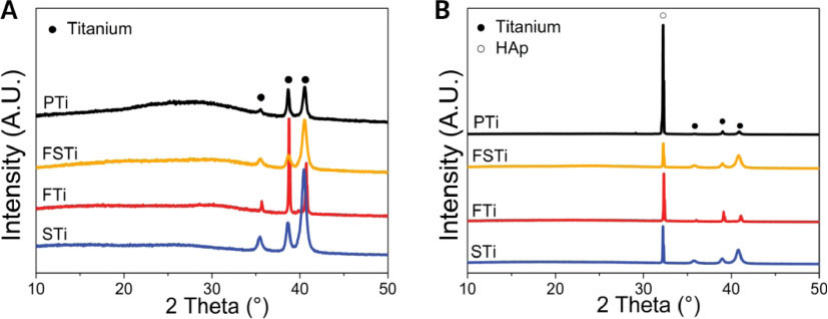
(**A**) Crystal structure of the titanium alloy surface detected by XRD. (**B**) XRD patterns of hydroxyapatite (HAp) deposition on the surface of samples in the different titanium alloy groups. Black dots represent characteristic peaks of titanium alloys, hollow dots represent HAp. The data were moved up equidistantly to reflect more information of the characteristic peaks and peak intensities brought about by the different processing methods. pTi: Polished titanium; STi: sandblasted titanium; FTi: femtosecond laser-treated titanium; FSTi: femtosecond laser-treated and sandblasted titanium.

XRD was the detection method used to understand the effect of sandblasting and femtosecond laser treatment on the titanium crystal structure. Figure 3A shows the XRD spectra (CuKa radiation at 40 kV/35 mA) of the titanium substrates before and after sandblasting and laser treatment. All of the samples yielded major α-phase titanium peaks, and all of the Ti samples presented no additional peaks of anatase titania (25.2° or 48°). Some differences were noted in the pTi, including an increased background area between 20 and 35°, which represented the amorphous phase of the sample. This indicated that many amorphous phases were present in the pTi. After sandblasting (STi), three sharper peaks could be observed at 35.4°, 38.6° and 40.5°, indicating crystalline α-phase titanium. Laser treatment resulted in the sharpest peaks and slightly shifted the peak position to a larger angle, which may have been caused by the dislocated areas constituting the surface roughness. Moreover, the titanium substrates showed weaker peaks after both sandblasting and laser treatment than after sandblasting or laser treatment alone.

### Bioactivity *in vitro*

**Figure 4.**
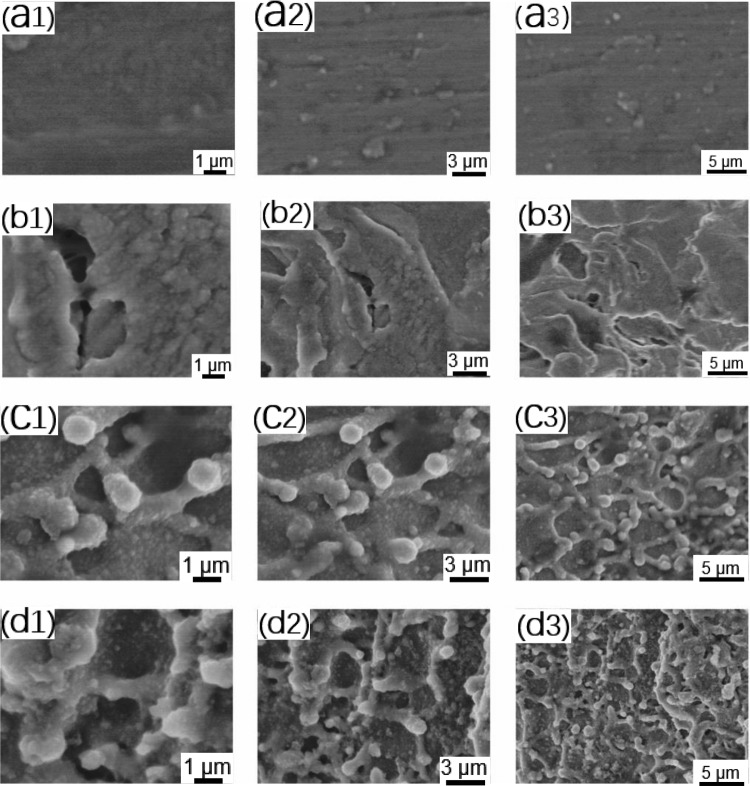
HAp Deposition on the surface of titanium alloys immersed in SBF imaged by SEM. (**A**) Polished titanium (pTi) at different magnifications; (**B**) sandblasted titanium (STi) at different magnifications; (**C**) femtosecond laser-treated titanium (FTi) at different magnifications; (**D**) femtosecond laser-treated and sandblasted titanium (FSTi) at different magnifications. The second and third columns in each group are low-magnification images of the samples.

To fully examine the effect of several sets of titanium morphologies on apatite deposition, SEM was used to observe the apatite deposition on the titanium alloy surface. As shown in Fig. 4, hydroxyapatite (HAp) formed on the surfaces of the titanium alloy samples after immersion. Unlike molten particles, HAp spheres are formed by the growth of crystal nuclei deposited on the surface of the pattern [30]. Figure 3B depicts the XRD spectra of HAp deposited on the surface of samples in each titanium alloy group after 14 days of incubation. The characteristic HAp peaks at 32° (211) were clearly observed in all samples, which aligns with the standard card no. 09-432 for HAp. No other peaks of the substrate were observed, revealing a dense coverage of HAp, which represents the preferred crystallographic orientation of the deposited HAp phase in the coating toward the (211) plane. Three titanium peaks were also detected. The intensity of the titanium peaks was much weaker than that of the HAp peaks. The intensity of the diffraction pattern is the only difference that was observed, and this difference was attributed to the different amounts of phases formed on each sample [31]. As seen, the characteristic peak intensity of HAp at 32 degrees (211) was pTi>FTi>STi>FSTi. A sharp increase in the characteristic peak intensity of HAp at 32° was observed for the polished titanium substrates, indicating an increase in the HAp deposition on these samples. The intensity of the 32° characteristic peak of the FSTi was the lowest, while that of the FTi was slightly higher than that of the STi. The formation of HAp on bioactive titanium in SBF is due to electrostatic interactions between the metal surface and ions in the fluid [32]. After immersion in SBF, the surface potential of the metal became very negative. As the immersion time increased, the surface potential increased to a maximum positive value and then decreased to a constant negative value.

### Protein adhesion assay

**Figure 5.**
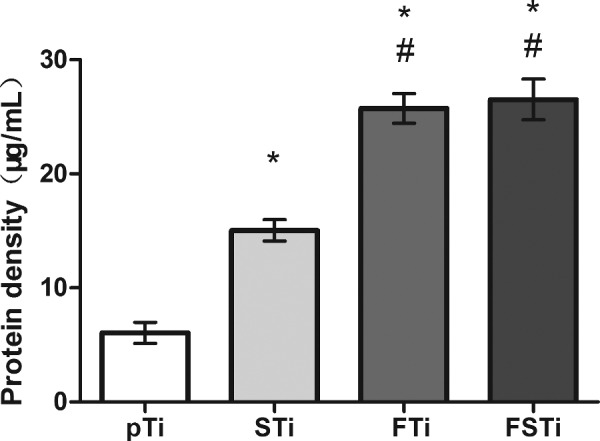
Protein adsorption to different specimens incubated in BSA. pTi: Polished titanium; STi: sandblasted titanium; FTi: femtosecond laser-treated titanium; FSTi: femtosecond laser-treated and sandblasted titanium significance: *P <* *0.05 (STi, FTi, FSTi vs pTi.); ^#^*P *<* *0.05 (pTi, FTi, FSTi vs STi).

Protein adhesion is regarded as an effective indicator of the biological activity of titanium implants. As shown in Fig. 5, the amount of protein adsorbed by the sandblasted titanium surface was higher than that of the pTi surface (*P *<* *0.05), and there was a significant difference between the two groups. The FTi samples had a more complex surface structure and specific surface area, and the amount of adsorbed protein was higher in the FTi group than in the pTi and STi groups (*P *<* *0.05). The protein deposition in the FSTi group was similar to that in the FTi group and was significantly different from that in the STi group.

### Cytotoxicity test

**Figure 6.**
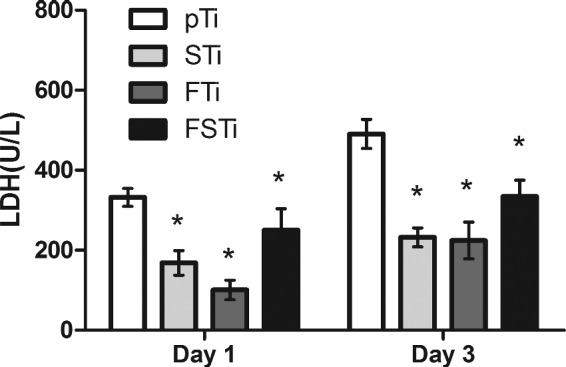
LDH Activity of osteoblasts on different titanium surfaces. pTi: Polished titanium; STi: sandblasted titanium; FTi: femtosecond laser-treated titanium; FSTi: femtosecond laser-treated and sandblasted titanium. Significance: *P <* *0.05 (compared with pTi).

It is necessary to detect the Al and V elements contained in titanium alloys and the potential cytotoxicity induced by the surface treatment of these alloys [33]. At the same time, the impact of melted particulates should also be considered. As shown in Fig. 6, the LDH content in the medium on day 1 was the highest in the pTi group and was significantly higher than that in the three other groups (*P *<* *0.05). After 3 days of culture, the highest level was still in the pTi group, with a significant difference compared with the other groups (*P *<* *0.05).

### Adhesion and proliferation

**Figure 7.**
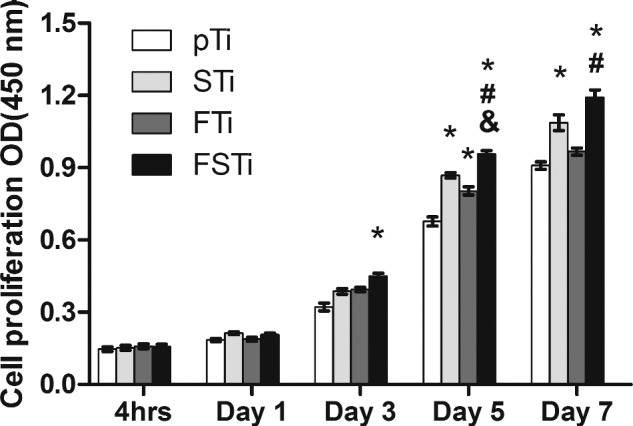
CCK-8 Assay to detect the osteoblast adhesion and proliferation on different titanium alloy surfaces. pTi: Polished titanium; STi: sandblasted titanium; FTi: femtosecond laser-treated titanium; FSTi: femtosecond laser-treated and sandblasted titanium. Significance: *P <* *0.05 (STi, FTi, FSTi vs pTi.); ^#^*P *<* *0.05 (FSTi vs STi) ; ^&^  *P *<* *0.05 (FSTi vs FTi).

The MC3T3-E1 cells were examined by CCK-8 assay. As shown in Fig. 7, after 4 h of incubation, there were no significant differences in cell adhesion among the four groups of titanium alloy surfaces. After 1 day of incubation, the differences among the groups were similar to those at 4 h, with only an increase in the number of cells in each group. After 3 days of incubation, the cells in each group showed a significant growth trend and differences among groups became obvious. Higher cell growth was observed in the FSTi group than in the pTi group, and the difference was significant (*P *<* *0.05). Although the number of cells in the STi and FTi groups increased, there was no significant difference compared with the pTi group. After 5 days of culture, each group maintained a continuous growth trend. The number of cells in the FTi and STi groups increased significantly compared with that in the pTi group (*P *<* *0.05). The FSTi group had the largest number of cells of all groups, and the differences were statistically significant (*P *<* *0.05). After 7 days of culture, the cell proliferation trend in each group was similar to that at 5 days. Only the difference between the STi group and the pTi group was reduced. The complex specific surface area and the suitable roughness had an effect on the osteoblasts that was beneficial for their proliferation.

### Osteoblast differentiation

**Figure 8.**
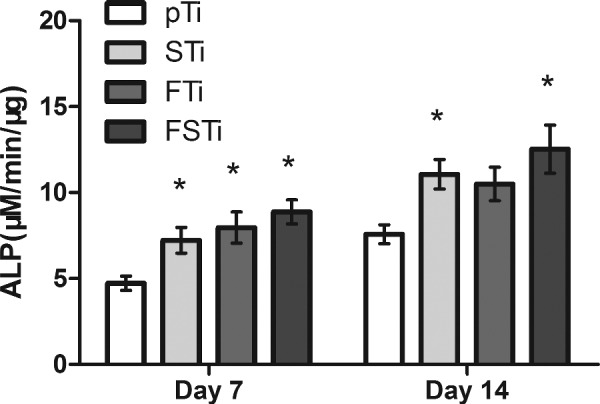
ALP Activity of osteoblasts on different titanium alloy surfaces. pTi: Polished titanium; STi: sandblasted titanium; FTi: femtosecond laser-treated titanium; FSTi: femtosecond laser-treated and sandblasted titanium. Significance: * *P *<* *0.05 (STi, FTi, FSTi vs pTi).

ALP activity was assessed to evaluate osteoblast differentiation. As shown in Fig. 8, after 7 days of culture, the ALP activity was significantly weaker in the pTi group than in the other groups (*P *<* *0.05). In other words, the osteoblasts in the STi, FTi and FSTi groups had better cell differentiation potential. Compared with the results at 7 days, the ALP activity in the pTi group increased progressively to 14 days of culture, that in the STi group increased slightly, and that in the FTi and FSTi groups showed a slower growth trend. Additionally, the difference between the STi and pTi groups was reduced. As osteoblasts differentiate into osteocytes, they gradually mineralize, and ALP activity begins to decrease.

### Cell morphology

**Figure 9.**
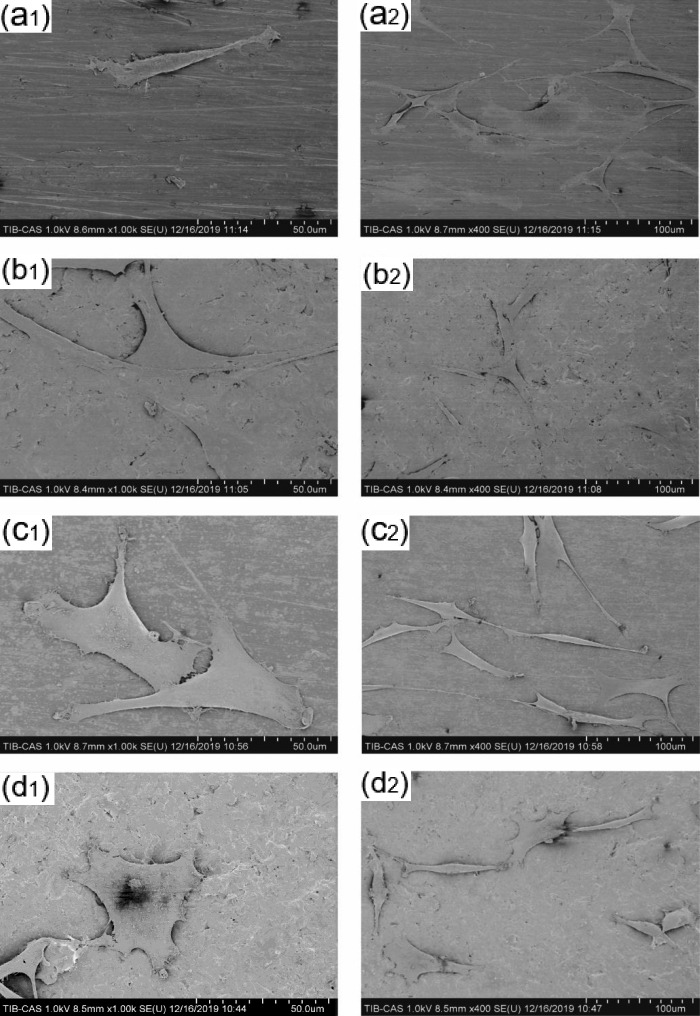
Spreading of osteoblasts on the surface of titanium alloys. (**A**) Polished titanium (pTi); (**B**) sandblasted titanium (STi); (**C**) femtosecond laser-treated titanium (FTi); (**D**) femtosecond laser-treated and sandblasted titanium (FSTi).

SEM was used to observe the spreading of the MC3T3-E1 cells on each titanium plate. As shown in Fig. 9, the osteoblasts in the pTi group showed a characteristic growth direction. The cells exhibited the protrusion of a small number of pseudopods around the surface and a spindle-like shape and were arranged in parallel on the polished surface. The osteoblasts in the STi group were also triangular, with pseudopod protrusion and microfilament extension. The osteoblasts in the FTi and FSTi groups showed a spindle-like shape and unoriented polygonal extension; this state also extended to the periphery of the cells, which presented slightly pronounced outstretched lamellipodia and filopodia.

### ECM mineralization assay

**Figure 10.**
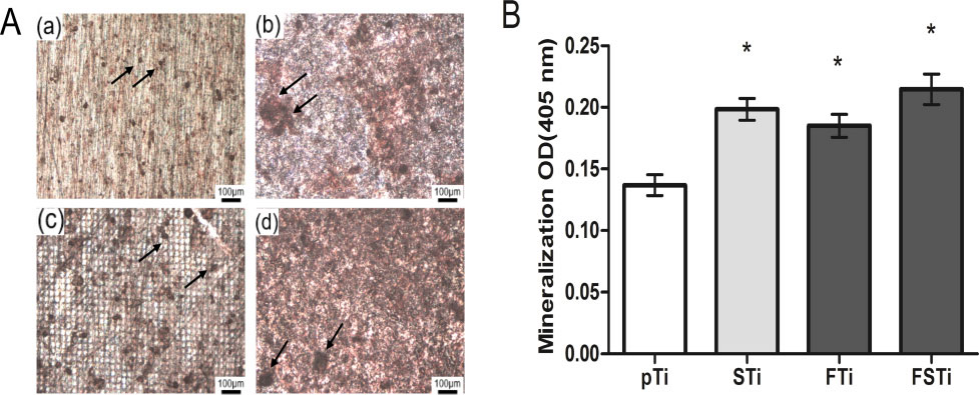
ECM Mineralization of samples stained after 21 days of culture with osteoblasts. (**A**) Qualitative analysis of osteoblasts cultured for 21 days (alizarin red stain, black arrows point to calcium nodules). (**B**) Quantitative analysis of ECM mineralization after 21 days of osteoblast culture. (a): polished titanium (pTi); (b): sandblasted titanium (STi); (c): femtosecond laser-treated titanium (FTi); (d): femtosecond laser-treated and sandblasted titanium (FSTi). Significance: *P < 0.05 (STi, FTi, FSTi vs pTi).

The mineralization ability of osteoblasts was determined by alizarin red S staining, which was carried out after 21 days of culture. As shown in Fig. 10A, there were a small number of mineralized calcium nodules in the pTi group, and the area of a single calcium nodule was small. In the STi group, there were a small number of mineralized calcium nodules. In the FTi group, a grid-like background appears in the field of view, which is the laser beam array remaining after femtosecond laser scanning. There were a large number of mineralized calcium nodules, and the area of a single calcium nodule was larger than that in the pTi group. In the FSTi group, there were a large number of mineralized calcium nodules, and the size of a single calcium nodule was larger in the FSTi group than in the FTi group. The quantitative analysis results are shown in Fig. 10B. The content of mineralized calcium nodules was significantly lower in the pTi group than in the other three groups (*P *<* *0.05).

### Collagen secretion

**Figure 11.**
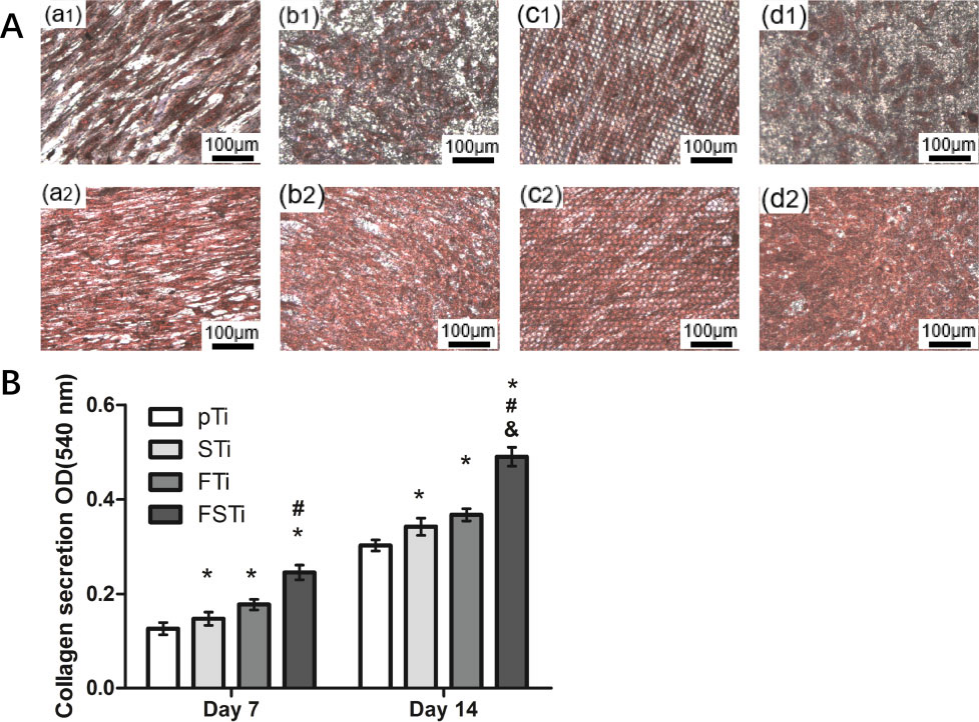
Collagen secretion after 7 and 14 days of osteoblast culture and staining by sirius red. (**A**) (a): Polished titanium (pTi); (b): sandblasted titanium (STi); (c): femtosecond laser-treated titanium (FTi); (d): femtosecond laser-treated and sandblasted titanium (FSTi). Qualitative analysis of osteoblasts cultured for 7 and 14 days; the first row shows osteoblasts stained after 7 days and the second row shows osteoblasts stained after 14 days. (**B**) Quantitative analysis of collagen after 7 and 14 days of osteoblast culture. Significance: *P <* *0.05 (STi, FTi, FSTi vs pTi.); ^#^*P *<* *0.05 (FSTi vs STi) ; ^&^*P *<* *0.05 (FSTi vs FTi).

Most collagen in the ECM is secreted by osteoblasts, so the level of collagen secretion is also considered an important manifestation of osteoblast growth. Collagen secretion was qualitatively analyzed by sirius red staining and imaging, as shown in Fig. 11A. When the pTi group was cultured for 7 days, the nuclei were densely stained and the cells stretched to a higher degree. The remaining three groups have a lower degree of extension. After 14 days of culture, the collagen of each group of cells basically filled the field of vision, and the overlap of cells in some areas showed extremely deep staining, with only a small amount of unfilled space. The level of collagen secretion was significantly lower in the pTi group than in the remaining three groups (Fig. 11B). The order of collagen secretion was FSTi > FTi > STi (*P *<* *0.05). After 14 days of incubation, the amount of collagen secreted on the titanium samples in each group increased, and the growth difference between the groups was the same as that on day 7.

### Macrophage polarization

**Figure 12.**
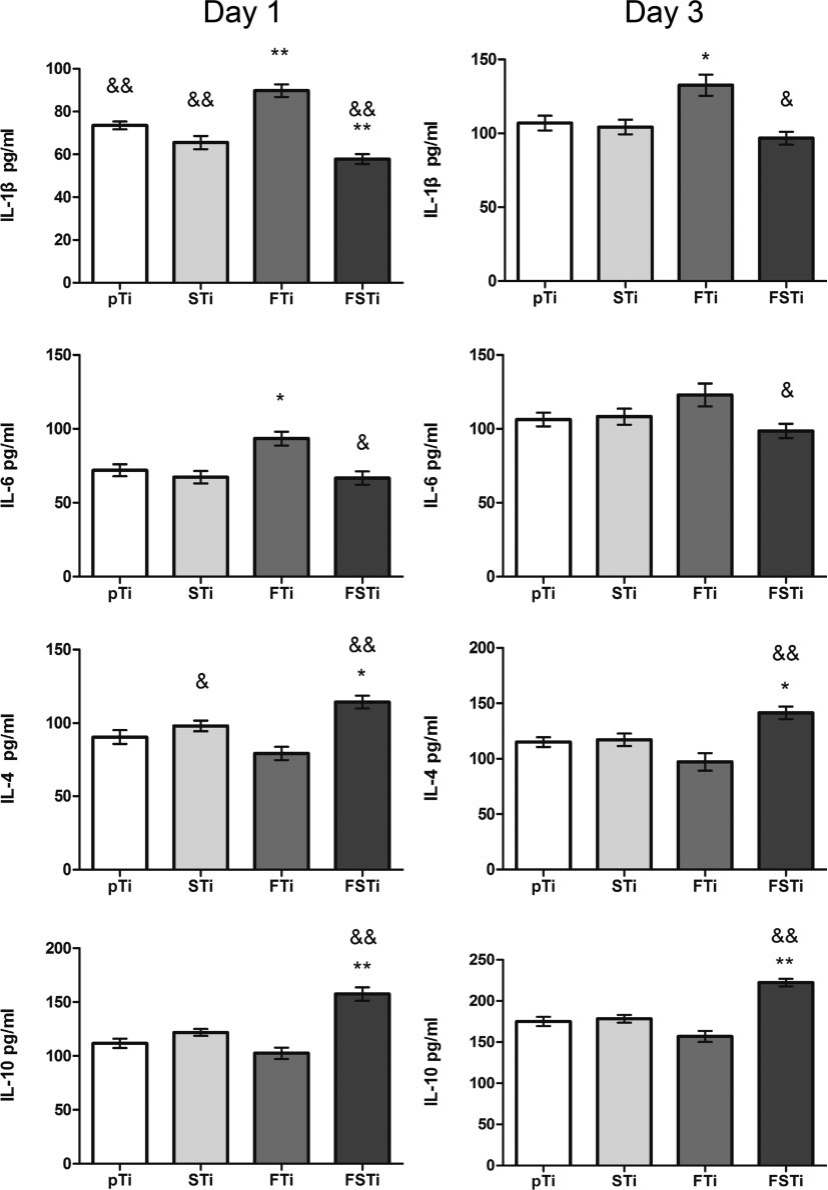
Inflammatory factor secretion of osteoclasts cultured on the sample surface for 1 and 3 days. pTi: polished titanium; STi: sandblasted titanium; FTi: femtosecond laser-treated titanium; FSTi: femtosecond laser-treated and sandblasted titanium. The left panel shows the release of inflammatory factors after 1 day of culture, and the right panel shows the release after 3 days. The parallel images indicate the same detection index. Significance: *P <* *0.05 (STi, FTi, FSTi, vs pTi); ^#^*P *<* *0.05 (pTi, FTi, FSTi, vs STi); ^&^*P *<* *0.05 (pTi, STi, FSTi, vs FTi).

The polarization of macrophages into the M1 and M2 subtypes occurs as part of the host response. In many cases, efficient and timely phenotypical transformation is essential for proper and functional reconstruction.[34] After 1 day of culture, the levels of proinflammatory and anti-inflammatory factors in the macrophage culture medium in each group were detected. As shown in Fig. 12, the level of the proinflammatory factor IL-1β in the hydrophobic nanoscale group (FTi) was significantly different from that in the pTi group (*P *<* *0.05) and the other three groups. Interestingly, only the hydrophilic microscale group and the hydrophilic micro-/nanostructure group showed lower levels of IL-1β secretion, which were significantly different from that of the hydrophobic nanoscale group (*P *>* *0.05). Regarding the secretion of IL-6, which is also a proinflammatory factor, compared with the pTi group, only the hydrophobic nanoscale group showed a significant difference (*P *<* *0.05). In contrast, the hydrophilic micro-/nanostructure group (FSTi) showed higher secretion levels of the anti-inflammatory factors IL-4 and IL-10. Compared with the hydrophobic nanoscale group (FTi), IL-4 secretion in the hydrophilic micro-/nanostructure group (FSTi) was significantly increased (*P *<* *0.05). IL-4 secretion in the hydrophilic microscale group (STi) was also higher than that in the hydrophobic nanoscale group (FTi) (*P *<* *0.05). Regarding IL-10 secretion, the FSTi group showed a significantly higher level than the other three groups (*P *<* *0.05).

After 3 days of culture, the levels of inflammatory factors were increased compared to their levels after only 1 day. The hydrophobic nanoscale group (FTi) showed a high level of proinflammatory IL-1β secretion that was significantly different from that in the hydrophilic polished group (pTi) (*P *<* *0.05). Notably, the difference between the hydrophobic nanoscale group (FTi) and the hydrophilic polished group (pTi) was reduced after 3 days compared with that on the first day. There were no significant differences in the IL-6 secretion levels among the groups. The levels of IL-4 and IL-10 secretion increased in all groups. The differences among the groups were similar to those detected on day 1.

## Discussion

As a useful processing technique, femtosecond laser treatment was chosen as an innovative technology for achieving texture with a precise topography. This technique has been applied in creating mastoid columns [35], laser-induced periodic surface structures (LIPSSs) [36] and (hybrid) micron/nanogrids [37]. The micrometer-scale roughness of Ti surfaces is one of the key parameters influencing osseointegration [38, 39]. A retrospective analysis of 252 articles by Souza et al. [40] identified 1–10 μm as the roughness range most suitable for bone implants. Wettability is considered to be another important surface property. The aluminum oxide used for sandblasting the surface of the initially slightly hydrophilic titanium alloy has a certain degree of hydrophilicity, and as the roughness increases, the wettability decreases, so the hydrophilic performance of the STi group is explained by the Wenzel model. At the same time, studies have shown that laser irradiation can generate many atomic forms of Al^3 +^ and O^2−^, among which aluminum atoms have only six electrons in the sp2^-^ hybrid orbital, which can easily form an eight-electron hydrophilic hydration structure with water molecules. It is also reported that a considerable number of unsaturated Al^3+^ and O^2−^ were not immediately paired, leading to dramatical growth of surface polarity [41]. The new laser ablated surface appears to be highly non-equilibrium. Therefore, FSTi group exhibits higher surface polarity, which leads to more hydrophilic behavior [42]. The wettability of the FTi group treated with femtosecond laser alone is consistent with other experimental reports [43]. Studies have shown that the high temperatures generated by laser burning can produce new crystal phases. The new crystal phase can cause differences in cell response. Femtosecond laser, as a cold cauterization technology, did not observe the production of new crystal phases in our inspections. Some other studies have shown that sandblasting and femtosecond laser treatment bring alumina impurities, nitrogen or the thickened oxide layer of amorphous TiO2 and the accumulated carbon compounds to the titanium alloy substrate [44–47]. However, the latter three types of pollution are unavoidable reactions in the treatment process. The aging process can change the wettability and affect the cell response. Sader et al. did not observe any detrimental effect of residual blasting alumina particles in an *in vitro* experiment using osteoblasts [48, 49]. Then the treated titanium alloy surface was put into deionized water for ultrasonic cleaning. At the same time, the laser-treated titanium alloy surface was stored and transported in vacuum. These measures are to reduce the influence of surface chemical impurities on the experimental results.

The biological activity of biomaterials is considered to be one of the important factors affecting the osseointegration performance of implant devices. SBF immersion tests have been identified to predict the relative *in vivo* bioactivity of biomaterials [50]. Some scholars believe that the adsorption of organic proteins in plasma can also reflect the biological activity of a device *in vivo* [51]. Therefore, when designing the experiment, we adopted two methods to detect protein adsorption and selected albumin, which is the protein with the highest content in plasma [52]. Similarly, some scholars have used fibronectin to verify that the results of the two proteins may be different or even opposite; these differences can arise from the different adsorption characteristics of the proteins [53]. Interestingly, the protein adsorption (Fig. 4) and HAp deposition results (Fig. 3B) were quite different in our experiments. The former suggested that the FSTi group had a better protein adsorption capacity. The properties and conformation of adsorbed proteins depend on the surface properties, protein properties and substrate retention time [54, 55]. There is a correlation between surface wettability and protein binding due to the presence of water molecules. At the same time, a larger specific surface area can provide more binding sites, which leads to the phenomena observed in the FSTi group. The deposition of HAp on bioactive titanium metal immersed in SBF is due to electrostatic interactions between the metal surface and ions in the fluid [32, 50]. The contact angle decreases as the charge stored on the surface increases [56]. Therefore, the FSTi group, with a lower water contact angle, or higher surface potential, should have shown greater HAp deposition. However, less HAp was deposited on the surface in the FSTi group than was expected based on the results of the SBF experiments. It may be that the HAp is only deposited on the surface, while the rest of the samples with surface structure can be deposited in the voids or gaps resulting in non-standard sampling. However, in other reports, femtosecond laser-modified surfaces have shown good HAp deposition capabilities [57]. The LDH assay was applied to assess the effect of molten metal on cytotoxicity and showed that the combination of femtosecond laser treatment and sandblasting increased cytotoxicity to some extent, but the cytotoxicity of the resulting surfaces was still lower than that of the polished titanium surface.

Well-defined multiscale structures are able to regulate the cellular morphology and function of bone-related cells, such as mesenchymal stem cells (MSCs), osteoblasts and osteoclasts, through biochemical and mechanical signals in a physiological environment [58]. Cells react to the surface and the surrounding 3D environment, mechanically linking to the ECM through focal adhesion complexes and adapt their shape accordingly [59]. Cellular elongation can induce stretching of the cytoskeleton and nucleus, while compression can significantly alter the local charge density and ion concentrations, potentially activating osmotically sensitive ion channels [60]. These events regulate the proliferation and differentiation processes after adhesion. At the same time, the higher roughness and hydrophilicity were also the reasons for the early cell proliferation in FSTi group; To assess the enhanced osseointegration capacity of the Ti substrates, the ALP activity and extent of ECM mineralization are widely used as markers of early and late differentiation of osteoblast-like cells [61, 62]. However, in our study, the ALP activity of osteoblasts had gradually decreased by day 14 in the FTi group, which may indicate that the ALP activity gradually decreased after reaching its maximum before remineralization [63]. The increase of ALP in STi and FSTi groups may be due to the promotion of roughness and wettability. The difference in wettability between the two may lead to higher ALP activity for the latter; in the ECM mineralization experiment, The FSTi group showed obvious calcium deposition and both the STi and FTi groups had certain advantages. These phenomena seem to indicate that both roughness and wettability promote the early osseointegration process of osteoblasts. The combination of topographic clues and wettability provided by base roughness significantly enhances the early differentiation and mineralization of osteoblasts process. The results show that the base topography cues can be combined with wettability to achieve the cumulative effect of osteogenic differentiation. Collagen is the main secretion product in the process of osteoblast culture. As the dominant expression of osteoblast proliferation, the secretion of collagen matrix continues the differential expression of cell proliferation on the surface of each group of titanium alloys; The formation of a collagen matrix contributes to osteoblast differentiation and matrix mineralization [64]. These surface features may exert synergistic effects on cell differentiation and mineralization by controlling intercellular communication and activating signaling pathways. In addition, the enhanced wettability of the constructed surface also contributes to the osteoblast response [65]. Our findings indicate that femtosecond laser-treated titanium surfaces that are additionally sandblasted can stimulate matrix formation and enhance osteoblast function.

It has been shown that the macrophage phenotype is a key determinant in the nature of the healing response *in vivo* after the implantation of biological materials [66]. The cellular response of macrophages to the same surface *in vivo* occurs before undifferentiated mesenchymal cells reach the wound/implant site, and this macrophage response has an impact on the subsequent osteogenic process of osteoprogenitors. Considering the differences in roughness and surface wettability between the experimental groups and the control group, we speculate that the macrophage-specific polarization response may be related to differences in surface characteristics. Yet, the relationship of surface wettability and roughness with macrophage polarization is not fully confirmed. However, previous studies have shown that the secretion of IL-4 and IL-10 chemokines in macrophages cultured on a hydrophilic rough titanium surface increases [67]. This performance is consistent with our performance in the FSTi group. However, the reason for the insignificant difference in the STi group may be the difference in wettability. The upregulation of anti-inflammatory factors was observed in cells cultured on the hydrophilic rough surface, suggesting M2 macrophage activation. The significant difference between the FSTi group and the FTi group may be due to the wetting performance; Wettability has a stronger effect than roughness on interleukin secretion, it may not be the only reason for the increase in anti-inflammatory factors [67, 68]. Therefore, these surface-specific reactions suggest that chemical effects may lead to unique genotypical responses and to eventual phenotypical responses through properly optimized surface properties that affect macrophage activation.

The chemical composition of the surface is indeed an important part of the surface performance and has a unique influence on the cell–material response. The aging process can change the wettability and affect the cell response. However, the response of cells to aging caused by short-term exposure to the air is not clear. And studies have shown that the micro/nano layered Ti surface has a certain degree of antiaging ability [69]. Therefore, in the experimental design stage, we mainly select surface morphology, roughness, wettability and crystal lattice for characterization. Finally, we still hope that the surface chemical analysis experiment will be added to the next experimental design.

## Conclusion

In this study, a titanium alloy surface with the ability to promote early osseointegration and with improved anti-inflammatory properties was successfully prepared. Through the combination of sandblasting and femtosecond laser treatment, a micro-/nanostructured with roughness and hydrophilic properties was obtained. The titanium substrate was bioactive, induced improved early osteoblast interactions and promoted macrophage polarization. The synergy of the two functions reduced the time required for early osseointegration and achieved better bone-titanium mechanical interlocking. Our study reveals the synergistic effect of a micro-/nanostructured structure and wettable surface on early bone healing and provides insights into the future design of optimal implant surfaces.
